# P-372. *Candida auris* Outbreak in a Cardiothoracic Transplant Intensive Care Unit: Implications for Infection Prevention

**DOI:** 10.1093/ofid/ofae631.573

**Published:** 2025-01-29

**Authors:** Shardul N Rathod, Grace Barajas, Brooks I Mitchell, Michael Malczynski, William Justin Moore, Erin Weslander, Christie M Bertram, Sarah Sutton, Valentina Stosor, Chao Qi, Teresa Zembower, Maureen K Bolon

**Affiliations:** Northwestern Memorial Hospital, Chicago, Illinois; Northwestern Memorial Hospital, Chicago, Illinois; Northwestern University Feinberg School of Medicine, Chicago, Illinois; Northwestern Memorial Hospital, Northwestern University Feinberg School of Medicine, Chicago, Illinois; Northwestern Medicine, Chicago, Illinois; Northwestern Memorial Hospital, Chicago, Illinois; Northwestern Memorial Hospital/Rosalind Franklin University of Medicine and Science, Chicago, Illinois; Northwestern University, Chicago, Illinois; Northwestern University Feinberg School of Medicine, Chicago, Illinois; Northwestern University Feinberg School of Medicine, Northwestern Memorial Hospital, Chicago, IL; Northwestern University, Chicago, Illinois; Northwestern University Feinberg School of Medicine, Chicago, Illinois

## Abstract

**Background:**

*Candida auris* is a yeast that causes invasive infections such as bloodstream infections (BSIs); mortality rates for *C. auris* BSIs may be as high as 80%. Rates have been climbing in the U.S. Exposure to skilled nursing facilities (SNFs), long-term acute care hospitals (LTACHs), or inpatient rehabilitation facilities (IRFs) represents a known risk factor for *C. auris*. Our institution experienced a *C. auris* outbreak from September 2023 - January 2024.

**Figure 1:**
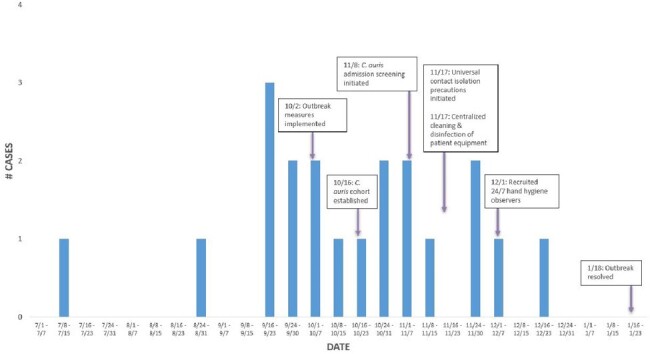
C. auris Outbreak Epidemic Curve

**Methods:**

*C. auris* cases in the outbreak were identified from clinical specimens and admission and weekly surveillance via polymerase chain reaction (PCR). Clinical characteristics were determined through chart review. A multidisciplinary team implemented infection prevention (IP) interventions to curb nosocomial spread.

**Figure:**
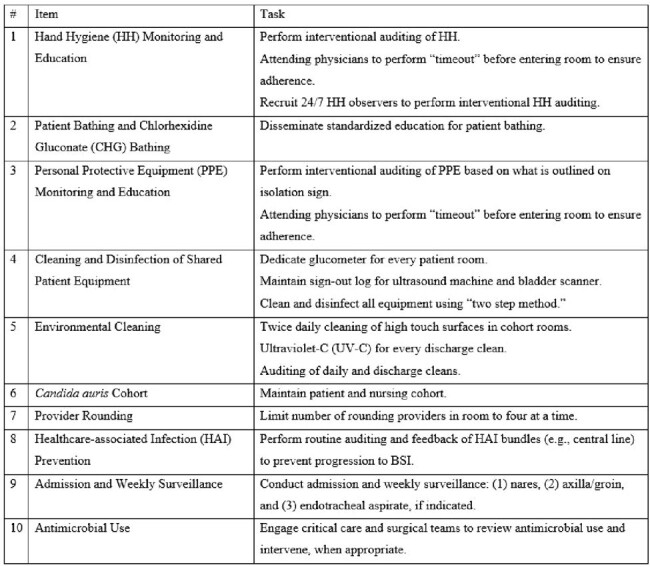

Infection Prevention Interventions

**Results:**

There were 20 cases of *C. auris* identified in our CTICU between July 2023 and January 2024 (Figure 1). Whole-genome sequencing and phylogenetic analysis based on pairwise single nucleotide polymorphism (WG-SNP) distance revealed two outbreak clusters.

Of the 20 patients, 13 (65%) were solid organ transplant/acute mechanical circulatory support patients and three (15%) had prior SNF, LTACH, or IRF exposure. Four (20%) patients had *C. auris* BSIs and seven (35%) patients died, including all patients with BSIs. The mean duration of antibiotic use in the four patients was 67 days.

IP interventions addressed potential vehicles of transmission including healthcare personnel hands, shared patient equipment, and the environment (Table 1).

**Conclusion:**

Only 15% of patients had prior SNF/LTACH/IRF exposure; many were admitted from home and/or outside hospitals with risk factors including recent surgery and antimicrobial use. This suggests the epidemiology of *C. auris* may be changing, and it may be prudent to consider routine admission and weekly surveillance on inpatient units deemed high risk for *C. auris*. Effective IP interventions included interventional hand hygiene auditing, increased accountability and oversight for shared patient equipment, limiting the number of rounding providers in rooms, and auditing environmental cleaning. Sustaining these efforts is crucial to keep pace with an evolving *C. auris* landscape.

**Disclosures:**

**All Authors**: No reported disclosures

